# Effectiveness and Safety of Simnotrelvir/Ritonavir and Nirmatrelvir/Ritonavir in the Treatment of Moderate to Severe COVID‐19

**DOI:** 10.1002/iid3.70174

**Published:** 2025-04-14

**Authors:** Xin Chen, Xiao‐Qing Lin, Fang Cheng, Shi‐Lin Zheng, Qiang Zhang, Te Wu, Xian‐Gao Jiang, Ji‐Chan Shi

**Affiliations:** ^1^ Department of Infectious Disease Wenzhou Central Hospital, Dingli Clinical College of Wenzhou Medical University, Wenzhou Sixth People's Hospital Zhejiang China

**Keywords:** ADR, clinical effectiveness, COVID‐19, nirmatrelvir/ritonavir, simnotrelvir/ritonavir, small‐molecule antiviral drugs

## Abstract

**Background and Aim:**

Simnotrelvir/ritonavir and nirmatrelvir/ritonavir are major treatments for COVID‐19, but their comparative efficacy and safety, especially in patients with moderate to severe COVID‐19, remain unclear.

**Methods:**

This was a retrospective cohort study using electronic medical record data. From May 30, 2023, to October 8, 2023, 115 patients with moderate to severe COVID‐19 were retrospectively collected from Wenzhou Central Hospital. They were treated with simnotrelvir/ritonavir or nirmatrelvir/ritonavir. The clinical effectiveness and adverse reactions were analyzed and compared between the two groups.

**Results:**

A total of 115 hospitalized patients were included in the study. They were 65 (56.5%) men and 50 (43.5%) women, with a mean age of 61 years. 58 (50.4%) were treated with simnotrelvir/ritonavir and 57 (49.6%) with nirmatrelvir/ritonavir. There was a similar rate of composite disease progression (10.3% vs. 7.0%, *χ* ^2^ = 0.401, *p* = 0.527) and mortality (5.2% vs. 3.5%, *χ* ^2^ = 0.191, *p* = 0.662) between the two groups. The progression rate from moderate COVID‐19 to severe COVID‐19 was not significantly different between the two groups (4.5% vs. 6.4%, *χ* ^2^ = 0.148, *p* = 0.701). Median time for hospitalization was 7.0 (6.0, 8.0) days and 9.0 (8.0, 10.0) days (*p* = 0.338), and time for SARS‐CoV‐2 negative conversion was 6.0 (6.0, 7.0) days and 7.0 (6.0, 7.0) days (*p* = 0.934) in the simnotrelvir/ritonavir group and nirmatrelvir/ritonavir group, respectively. Among moderate patients, time for hospitalization was shorter in the simnotrelvir/ritonavir group [6.0 (6.0, 7.0) vs. 8.0 (8.0, 10.0) days, log‐rank *p* = 0.004, HR = 1.838 (95% CI 1.199–2.815)]. And 5 (8.6%) had adverse drug reactions (ADRs) in the simnotrelvir/ritonavir group and 6 (10.5%) had ADRs in the nirmatrelvir/ritonavir group.

**Conclusion:**

This is the first study comparing the effectiveness of simnotrelvir/ritonavir and nirmatrelvir/ritonavir in moderate and severe COVID‐19 patients. Patients who received simnotrelvir/ritonavir exhibited shorter hospitalization. Disease progression, viral clearance times, and symptom resolution time were similar between the two groups.

## Graphical abstract

This is the first study comparing the effectiveness of simnotrelvir/ritonavir and nirmatrelvir/ritonavir in treating moderate and severe COVID‐19 patients. Patients who received simnotrelvir/ritonavir exhibited shorter hospitalization. Disease progression, viral clearance times, and symptom resolution time were similar between the two groups.

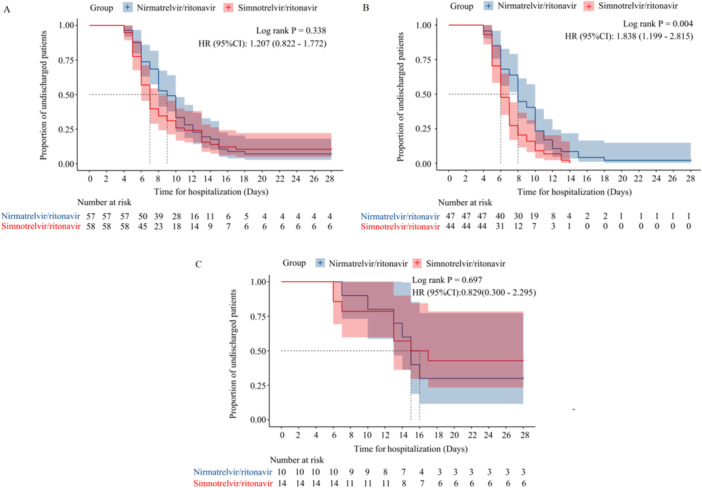

## 1 Introduction

Since December 2019, the world has been grappling with the severe acute respiratory syndrome coronavirus 2 (SARS‐CoV‐2) pandemic, which spreads among humans through respiratory and contact transmission, leading to the outbreak of coronavirus disease 2019 (COVID‐19). According to the official website of the World Health Organization (WHO), as of February 4, 2024, there have been over 774 million confirmed cases of COVID‐19 worldwide, with more than 7 million deaths [1], posing a huge threat to global public health. As of 2024, the Omicron variant, the fifth‐generation variant of SARS‐CoV‐2, is predominant and exhibits heightened infectivity and stronger antibody evasion [2, 3, 4]. Some patients with severe COVID‐19 can develop acute respiratory distress syndrome (ARDS) and systemic inflammatory response syndrome (SIRS). The activation of immune cells triggers an inflammatory cascade, which ultimately leads to multiple organ failure, with a fatality rate of as high as 49% [5]. Some immunocompromised patients may not be able to produce an adequate immune response after receiving the COVID‐19 vaccine [6]; antiviral drugs are critical for these patients. 3CL protease (3CLpro) is an established target for anti‐coronavirus drugs [7], and 3CL protease inhibitors can prevent SARS‐CoV‐2 replication, thereby accelerating virus clearance and reducing the chance of “cytokine storms.” Therefore, there is an urgent need for new antiviral drugs for COVID‐19.

At present, the small‐molecule antiviral drugs approved in China include nirmatrelvir/ritonavir, molnupiravir, azvudine, simnotrelvir/ritonavir, and mindeudesivir. Nirmatrelvir/ritonavir is recommended by the National Health Commission of China guidelines for the treatment of mild to moderate COVID‐19 adult patients with risk factors for progression to severe disease [8]. On March 1, 2023, simnotrelvir/ritonavir was approved by the National Medical Products Administration and recommended by the Diagnosis and Treatment Protocol for COVID‐19 patients (Tentative 10th Version) for the treatment of adult patients with mild to moderate COVID‐19 [9].

Nirmatrelvir/ritonavir has been shown to reduce the risk of hospitalization and mortality in outpatients with mild to moderate disease in the early stages of the disease [10, 11, 12]. However, there is limited clinical evidence of efficacy in hospitalized patients with moderate to severe COVID‐19. A phase II/III clinical study [13] evaluated the efficacy and safety of simnotrelvir in adult patients with mild to moderate COVID‐19. In this study, the time to sustained resolution of COVID‐19 symptoms was significantly shorter in the simnotrelvir group than in the placebo group by a median of 35.8 h. Besides, on Day 5, the reduction of viral load from baseline after treatment with simnotrelvir was significantly decreased in the simnotrelvir group compared to the placebo group [13]. However, there was a lack of data on effectiveness in patients with severe COVID‐19. Relevant studies are lacking to compare the efficacy of simnotrelvir/ritonavir with nirmatrelvir/ritonavir in improving clinical outcomes in patients with moderate to severe COVID‐19.

Our hospital (Wenzhou Central Hospital, Zhejiang Province, China) is a designated hospital for COVID‐19 admission in Wenzhou City, and antiviral therapy with simnotrelvir/ritonavir has been used in some hospitalized and outpatients with COVID‐19 since May 2023. To compare the effectiveness and safety of simnotrelvir/ritonavir and nirmatrelvir/ritonavir in patients with moderate to severe COVID‐19, we retrospectively analyzed COVID‐19 patients who were hospitalized in our hospital from May 30, 2023, to October 8, 2023.

## 2 Materials and Methods

### 2.1 Study Subjects and Inclusion Criteria

A total of 115 hospitalized COVID‐19 patients who received simnotrelvir/ritonavir or nirmatrelvir/ritonavir and were admitted to this hospital between May 30, 2023, and October 8, 2023, were retrospectively included. Inclusion criteria: (1) ≥ 18 years of age; (2) patients with a positive SARS‐CoV‐2 test and a confirmed diagnosis of COVID‐19; (3) the presence of one or more COVID‐19 symptoms, such as fever, cough, sore throat, headache, muscle pain, nausea, vomiting, diarrhea, and shortness of breath on exertion; and (4) at least one of them (imaging showing lung involvement or SpO_2_ ≤ 93% or the need for oxygen or mechanical ventilation). Exclusion criteria: (1) ALT or AST higher than 5 times the upper limit of normal; (2) estimated glomerular filtration rate (eGFR) < 30 mL/min (including patients receiving hemodialysis or hemofiltration); (3) expected discharge within 72 h or transfer to another hospital that is not the study site; (4) current or expected use of highly dependent CYP3A4 enzyme clearance or strong CYP3A4 enzyme inducer of any drug or substance; and (5) pregnant or lactating women. COVID‐19 diagnostic criteria and clinical staging were based on the “Diagnosis and treatment protocol for COVID‐19 patients (Tentative 10th Version)” [8]. The retrospective cohort data were obtained from the electronic medical record. This study was approved by the Ethics Committee of Wenzhou Central Hospital (L2023‐04‐079), and all patients were informed and agreed to participate in this study. This study included some moderate COVID‐19 patients who did not require oxygen therapy because these patients expressed their willingness to receive oral small‐molecule antiviral drugs during the outpatient recruitment stage and were admitted to the hospital for the convenience of full‐process management, so there was no crowding out of medical resources.

### 2.2 Treatment Programs

According to different treatments, 115 patients were divided into two groups. In the simnotrelvir/ritonavir group (*n* = 58), 750 mg of simnotrelvir and 100 mg of ritonavir were administered every 12 h for 5 days. In the nirmatrelvir/ritonavir group (*n* = 57), 300 mg of nirmatrelvir and 100 mg of ritonavir were administered every 12 h for 5 days or less. Since the data collection was based on the sampling, to ensure the validity and reliability of the study, the minimum sample size was computed. The sample size was determined by using the G*Power software (v3.1.9.7, Heinrich Heine Universität Düsseldorf, Düsseldorf, Germany). We used two‐sided testing, effect size = 0.8, *α* error probability = 0.05, and power (1−*β* error probability) = 0.95. The minimum sample size was computed to be 44 for each group and 88 for both groups overall. The number of individuals included in our study met the minimum sample size requirement, indicating some overall representativeness. The dose and course of treatment are recommended according to the drug instructions. Patients who were unable to tolerate oral administration received nasal feeding. All patients in both groups received bed rest and nutritional support. Some patients were given oxygen and symptomatic treatment based on the specific needs of each patient's condition. When combined with other infections, antibacterial drugs were incorporated into the treatment plan as deemed necessary. Regarding immunotherapy, all patients with severe COVID‐19 received corticosteroids (methylprednisolone). Besides, the administration of the drugs was promptly discontinued if intolerance or adverse reactions occurred.

### 2.3 Clinical Baseline Indicators

The demographic characteristics and clinical profiles of the whole analyzed population in the two groups were compared. This encompassed age, sex, vaccination status, the severity of COVID‐19, time from the onset of symptom to the first dose of medication, time from the first positive detection of SARS‐CoV‐2 by reverse transcription PCR (RT‐PCR) or rapid antigen method (RAM) to the first dose of medication, nucleic acid CT value by nasal swab, and COVID‐19 total score of related symptoms (scoring method: presence or absence of cough, nasal congestion/runny nose, sore/dry throat, shortness of breath/difficulty in breathing, malaise/fatigue, feeling hot/feverish, chills/shivering, muscle/body aches/headache, and nausea, each item is rated as 3 for severe, 2 for moderate, 1 for mild, and 0 for none; presence or absence of vomiting, diarrhea, loss of taste, or loss of smell, each item is rated as 3 for 5 times or more, 3 for 3 times to 4 times, and 1 for 1 time) (2 points for 5 or more episodes, 2 points for 3 to 4 episodes, 1 point for 1 to 2 episodes, and 0 points for none; the sum of all the scores is the total COVID‐19‐related symptom score, in which the severity of the symptom should be the most severe in the past 24 h) [13]. COVID‐19 high‐risk factors included age ≥ 65 years, cardiovascular disease (including hypertension), chronic lung disease, diabetes mellitus, chronic liver disease, renal disease and maintenance dialysis patients, tumors and other underlying diseases, immune deficiency (e.g., AIDS patients and chronic use of corticosteroids or other immunosuppressive drugs leading to a state of immunocompromised function), obesity (BMI > 30), and heavy smokers.

### 2.4 Outcome Indicators

Primary outcome indicators: (1) compound disease progression, which was defined as any of the following events: (i) noninvasive respiratory support; (ii) initiation of endotracheal intubation; (iii) intensive care unit admission; and (iv) all‐cause death within 28 days; a patient with the occurrence of any of the above events was defined as having compound disease progression; and (2) progression of moderate COVID‐19 to severe and critical COVID‐19 [moderate COVID‐19 defined as meeting any one of the following: (i) persistent high fever > 3 days or (and) cough and shortness of breath, but respiratory rate (RR) < 30 breaths/min and oxygen saturation > 93% on air inhalation at rest; (ii) characteristic pneumonic manifestations of COVID‐19 infection can be seen on imaging; severe COVID‐19 defined as meeting any one of the following: (i) shortness of breath, RR ≥ 30 times/min; (ii) in the resting state, the pulse oxygen saturation (SpO_2_) is ≤ 93% while breathing ambient air; (iii) arterial partial pressure of oxygen (paO_2_)/the fraction of inspired oxygen (FiO_2_) ≤ 300 mmHg; (iv) the clinical symptoms are progressively worse, and lung imaging shows that the lesion has progressed significantly > 50% within 24–48 h; critical COVID‐19 defined as meeting any one of the following conditions: (i) respiratory failure and mechanical ventilation; (ii) shock; and (iii) ICU admission due to other organ failures].

Secondary outcome indicators: (1) time for hospitalization. Time for hospitalization was referred to the “Diagnosis and treatment protocol for COVID‐19 patients (Tentative 10th Version)” [8]. Discharge criteria: significant improvement in condition, stable vital signs, normal body temperature for more than 24 h, lung imaging showed significant improvement in acute exudative lesions and could be switched to oral medication without complications that required further management, and other conditions. (2) Time for SARS‐CoV‐2‐negative conversion (defined as SARS‐CoV‐2 antigen‐negative or nucleic acid cycle threshold [CT] values of ≥ 35).

Adverse drug reactions (ADRs) Included allergic reactions (itching, rash, etc.), gastrointestinal symptoms (nausea, vomiting, etc.), and abnormal laboratory indicators (decreased neutrophil count, abnormal blood lipids, hyperuricemia, elevated transaminases, elevated serum creatinine, elevated blood creatine kinase, etc.).

### 2.5 Statistical Methods

R.4,3,1 software was used for data analysis. The G*Power 3.1.9.7 software was used to estimate the minimum sample size. The nonnormal distribution data were represented as median (P25, P75), and the nonparametric rank‐sum test (Mann–Whitney *U* test) was used for comparison between groups. Count data were expressed as *n* (%), and comparisons between groups were made using *χ* ^2^ test or Fisher's exact text. Comparisons of clinical recovery time within 28 days and clinical recovery time within 28 days were made using Kaplan–Meier method. Comparisons of time for hospitalization and time for SARS‐CoV‐2‐negative conversion within 28 days were made using the Kaplan–Meier method. *p* < 0.05 indicated that the difference was statistically significant.

## 3 Results

### 3.1 Clinical Baseline Characteristics and Group Comparison

The study included a total of 115 hospitalized patients. There were 65 (56.5%) men and 50 (43.5%) women. The mean age was 61 years, ranging from 18 to 96 years. As shown in Table 1, 115 patients, including 58 in the simnotrelvir/ritonavir group and 57 in the nirmatrelvir/ritonavir group, were included. The differences in sex, age, vaccination, COVID‐19 severity, COVID‐19‐related symptom scores, high‐risk factors, time from detection of SARS‐CoV‐2 positivity to the first dose of medication, and CT values of nucleic acids via nasal swabs were not statistically significant between the two groups (all *p* > 0.05), indicating that the baseline is comparable between the two groups. The normality test results of continuous variables are shown in Table S1. Results suggested that all continuous variables do not conform to normal distribution. The distribution and comparison of COVID‐19 high‐risk factors between the two groups are shown in Table S2. The follow‐up period was 28 days.

**Table 1:** Clinical baseline characteristics.

Characteristics	Simnotrelvir/ritonavir group (*N* = 58)	Nirmatrelvir/ritonavir group (*N* = 57)	*Z*/*χ* ^2^	*p* value
Sex, *n* (%)			0.087	0.768
Male	32 (55.2)	33 (57.9)		
Female	26 (44.8)	24 (42.1)		
Age, median year (P25, P75)	67 (53.8, 76.5)	68 (35.5, 80.5)	−0.414	0.679
Number of vaccine doses			5.106	0.078
Unvaccinated	5 (8.6)	11 (19.3)		
Full vaccination+	52 (89.7)	42 (73.7)		
Booster vaccination++	1 (1.7)	4 (7.0)		
Severity of COVID‐19, *n* (%)			0.757	0.384
Moderate	44 (75.9)	47 (82.5)		
Severe	14 (24.1)	10 (17.5)		
Number of high‐risk factors			3.888	0.062
≤ 1	26 (44.8)	36 (63.2)		
≥ 2	32 (55.2)	21 (36.8)		
Time from confirmed SARS‐CoV‐2 to medication, median day (P25, P75)	0.0 (0.0, 1.0)	0.0 (0.0, 2.0)	−1.145	0.252
CT value at baseline, median (P25, P75)	29.8 (24.9, 31.5)	26 (20.9, 32.7)	−1.248	0.212
ORF1‐a/b gene, median (P25, P75)	30.0 (24.9, 31.8)	27 (21.9, 33.5)	−0.808	0.419
N gene, median (P25, P75)	29.9 (25.0, 31.7)	26 (20.9, 33.3)	−1.167	0.243
Time from symptom onset to medication, median day (P25, P75)	2.5 (1.0, 5.0)	5.0 (3.0, 6.0)	−3.45	< 0.001
Symptom scores	5.0 (4.0, 7.0)	5.0 (4.0, 6.0)	−0.303	0.762

^+^ Full vaccination indicates patients received two doses.

^++^ Booster vaccination indicates patients received three doses.

### 3.2 Comparison of Primary Outcome Indicators

The primary outcome indicators are summarized in Table 2. In total, the composite disease progression rate was 10.3% in the simnotrelvir/ritonavir group and 7.0% in the nirmatrelvir/ritonavir group. There was no significant difference between the two groups (*χ* ^2^ = 0.401, *p* = 0.527). The simnotrelvir/ritonavir group and nirmatrelvir/ritonavir group exhibited no statistically significant difference in the mortality rate (5.2% vs. 3.5%, *χ* ^2^ = 0.191, *p* = 0.662). In addition, no significant difference was found in composite disease progression rate and mortality rate for patients with different disease severities (*p* > 0.05). Regarding the rate of progression to severe COVID‐19 for patients with moderate COVID‐19, there were two (4.5%) patients in the simnotrelvir/ritonavir group and three (6.4%) patients in the nirmatrelvir/ritonavir group. This difference was not statistically significant (*χ* ^2^ = 0.148, *p* = 0.701). There was no statistical difference in the distribution of composite disease progression events between the simnotrelvir/ritonavir and nirmatrelvir/ritonavir groups (all *p* > 0.05) (Table 3).

**Table 2:** Primary outcome indicators in the simnotrelvir/ritonavir group and nirmatrelvir/ritonavir group.

	Simnotrelvir/ritonavir group	Nirmatrelvir/ritonavir group	*χ* ^2^	*p*
Moderate COVID‐19 (*n*)	44	47		
Progression to severe (*n*, %)	2 (4.5)	3 (6.4)	0.148	0.701
Death (*n*, %)	0 (0)	0 (0)	/	/*
Composite disease progression (*n*, %)	0 (0.0)	1 (2.1)	/	1.000#
Severe and critical COVID‐19 (*n*)	14	10		
Death (*n*, %)	3 (21.4)	2 (20.0)	0.007	0.932
Composite disease progression (*n*, %)	6 (42.9)	3 (30.0)	0.411	0.521
Total (*n*)	58	57		
Death (*n*, %)	3 (5.2)	2 (3.5)	0.191	0.662
Composite disease progression (*n*, %)	6 (10.3)	4 (7.0)	0.401	0.527

* No measures of association are computed for the crosstabulation of end point* group.

^#^ Fisher's exact test is used to compare the crosstabulation of end point* group.

**Table 3:** Distributions of composite disease progression events in the simnotrelvir/ritonavir group and nirmatrelvir/ritonavir group.

Total (*N* = 115)		*P*	Moderate COVID‐19 (*N* = 91)		*P*	Severe and critical COVID‐19 (*N* = 24)		*p*
*n* (%)			*n* (%)			*n* (%)		
Simnotrelvir/ritonavir group (*N* = 58)	Nirmatrelvir/ritonavir group (*N* = 57)		Simnotrelvir/ritonavir group (*N* = 44)	Nirmatrelvir/ritonavir group (*N* = 47)		Simnotrelvir/ritonavir group (*N* = 14)	Nirmatrelvir/ritonavir group (*N* = 10)	
(1) Noninvasive respiratory support								
0 (0.0)	1 (1.8)	0.496	0 (0.0)	0 (0.0)	/	0 (0.0)	1 (10.0)	0.417
(2) Initiation of tracheal intubation								
1 (1.7)	3 (5.3)	0.364	0 (0.0)	1 (2.1)	1.000	1 (7.1)	2 (20.0)	0.550
(3) Intensive care unit admission								
3 (5.2)	2 (3.5)	1.000	0 (0.0)	1 (2.1)	1.000	3 (21.4)	1 (10.0)	0.615
(4) Death								
3 (5.2)	2 (3.5)	1.000	0 (0.0)	0 (0.0)	/	3 (21.4)	2 (20.0)	1.000

*Note:* Fisher's exact test is used to compare the crosstabulation of end point* group.

### 3.3 Comparison of Secondary Outcome Indicators

**Figure 1:**
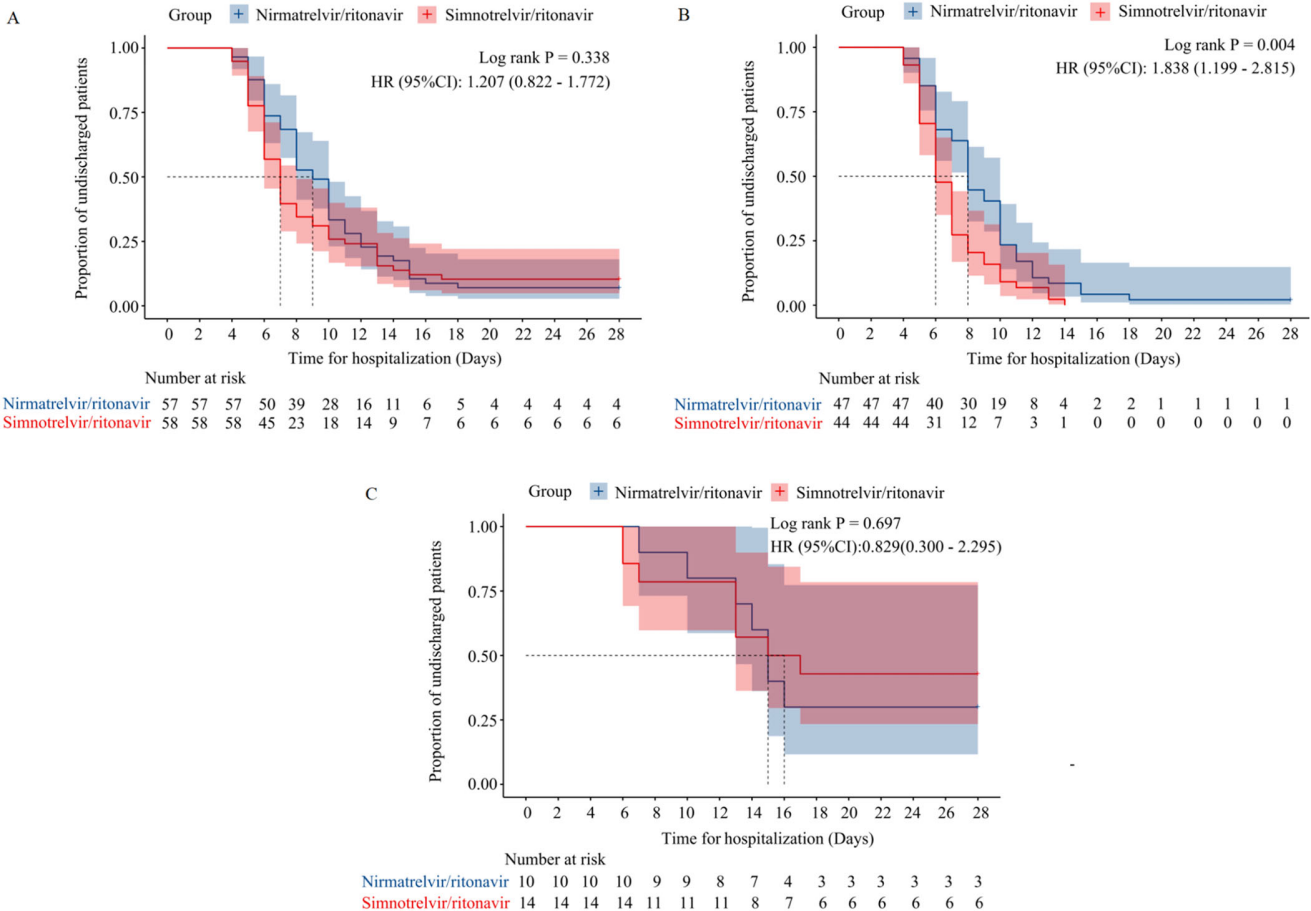
Kaplan–Meier curve of time for hospitalization in the simnotrelvir/ritonavir group and nirmatrelvir/ritonavir group. (A) Kaplan–Meier curve of time for hospitalization in the simnotrelvir/ritonavir group and nirmatrelvir/ritonavir group of total patients. (B) Kaplan–Meier curve of time for hospitalization in the simnotrelvir/ritonavir group and nirmatrelvir/ritonavir group of moderate COVID‐19 patients. (C) Kaplan–Meier curve of time for hospitalization in the simnotrelvir/ritonavir group and nirmatrelvir/ritonavir group of severe and critical COVID‐19 patients.

Median time for hospitalization was 7.0 (6.0, 8.0) and 9.0 (8.0, 10.0) days for patients in the simnotrelvir/ritonavir group and the nirmatrelvir/ritonavir group, respectively; log‐rank *p* = 0.338; HR = 1.207 (95% CI, 0.822–1.772) (Figure 1A). As shown in Figure 1B, among moderated COVID‐19 patients, time for hospitalization was significantly shorter in the simnotrelvir/ritonavir group than in the nirmatrelvir/ritonavir group [6.0 (6.0, 7.0) vs. 8.0 (8.0, 10.0) days, log‐rank *p* = 0.004, HR = 1.838 (95% CI, 1.199–2.815)]. In severe and critical COVID‐19 patients, there was no significant difference in time for hospitalization between the two groups (log‐rank *p* = 0.697) (Figure 1C).

**Figure 2:**
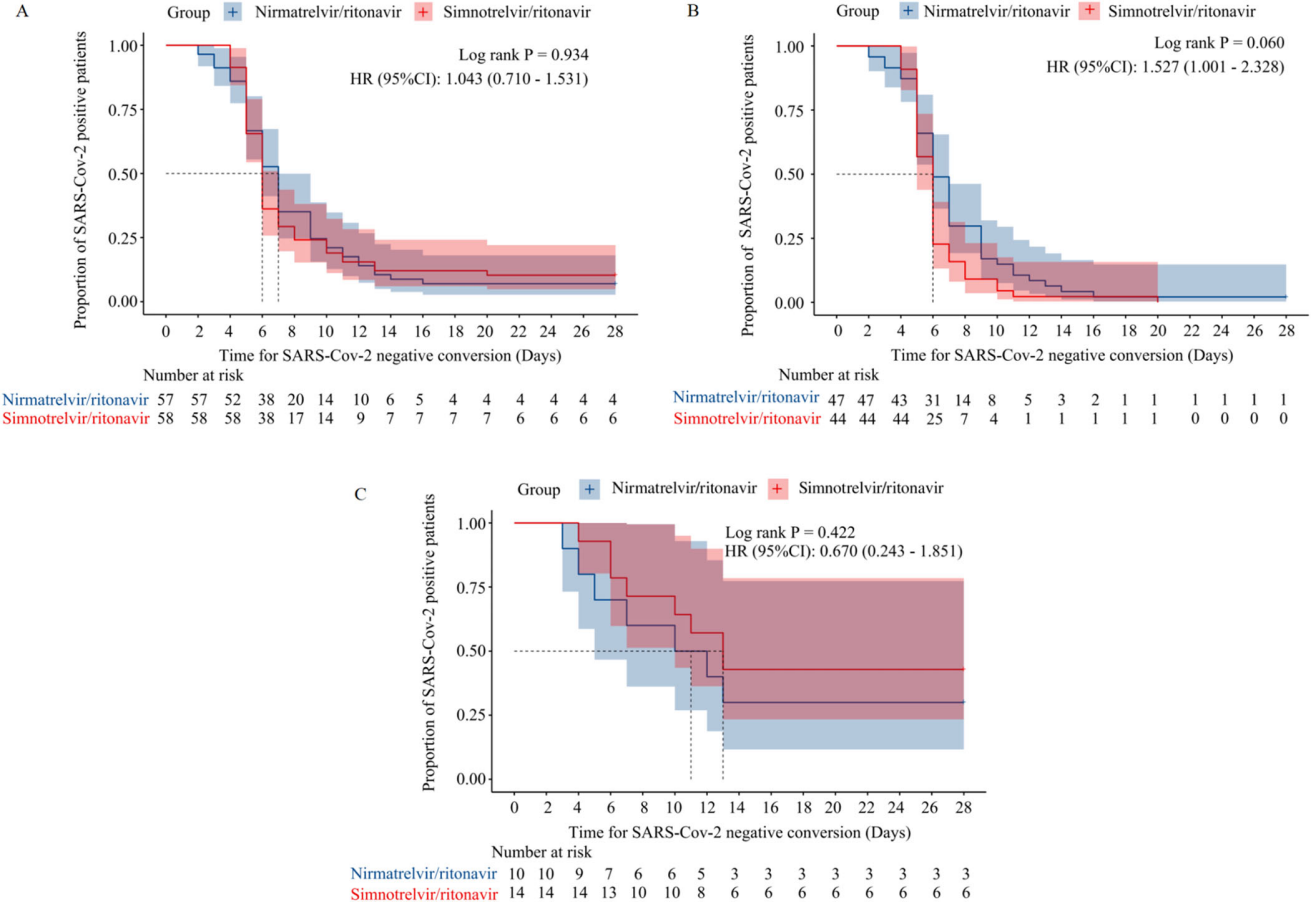
Kaplan–Meier curve of time for SARS‐CoV‐2‐negative conversion in the simnotrelvir/ritonavir group and nirmatrelvir/ritonavir group. (A) Kaplan–Meier curve of time for SARS‐CoV‐2‐negative conversion in the simnotrelvir/ritonavir group and nirmatrelvir/ritonavir group of total patients. (B) Kaplan–Meier curve of time for SARS‐CoV‐2‐negative conversion in the simnotrelvir/ritonavir group and nirmatrelvir/ritonavir group of moderate COVID‐19 patients. (C) Kaplan–Meier curve of time for SARS‐CoV‐2‐negative conversion in the simnotrelvir/ritonavir group and nirmatrelvir/ritonavir group of severe and critical COVID‐19 patients.

Time for SARS‐CoV‐2 negative conversion was 6.0 (6.0, 7.0) and 7.0 (6.0, 7.0) days, respectively, log‐rank *p* = 0.934, HR = 1.043 (95% CI, 0.710–1.531) (Figure 2A). Furthermore, no significant difference was found in time to negative conversion for patients with different disease severities (log‐rank *p* > 0.05) (Figure 2B–C).

### 3.4 Comparison of ADRs

**Figure 3:**
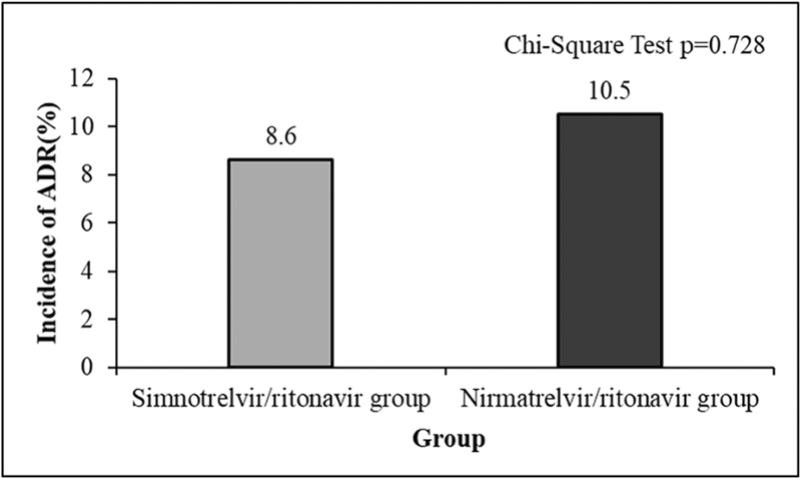
Comparison of the incidence of ADR in the simnotrelvir/ritonavir group and nirmatrelvir/ritonavir group.

Patients in both groups were regularly rechecked for blood routine and biochemical indexes, and ADRs were collected. 5 cases (8.6%) of ADRs occurred in the simnotrelvir/ritonavir group, including 1 case (1.7%) of elevated creatinine, 2 cases (3.4%) of mildly elevated aminotransferase (ALT < 2 times ULN), 1 case (1.7%) of hypertriglyceridemia, and 1 case (1.7%) of hyperuricemia. All these cases improved with symptomatic treatment, and the antiviral drug was not discontinued during the period. In the nirmatrelvir/ritonavir group, 6 cases (10.5%) of adverse reactions occurred, including 3 cases (5.3%) of transaminase elevation (ALT elevation > 2 times ULN) and 2 cases (3.5%) of drug allergic reactions (rash and itching), all of which improved after symptomatic treatment, and the antiviral drugs were not discontinued during the period. 1 case (1.8%) stopped drag administration due to a significant increase in creatinine. Figure 3 suggested that the rate of ADRs between the two groups was similar (*χ* ^2^ = 0.121, *p* = 0.728).

## 4 Discussion

Simnotrelvir/ritonavir is an oral small‐molecule antiviral that is a combination package consisting of simnotrelvir tablets (375 mg) and ritonavir tablets (100 mg) for the treatment of adult patients with mild to moderate COVID‐19. Simnotrelvir, like nirmatrelvir, is a SARS‐CoV‐2 coronavirus main proteinase (3CLpro) inhibitor that blocks viral replication by inhibiting viral 3CLpro so that it cannot process multiprotein precursors [14, 15, 16]; ritonavir is a strong inhibitor of cytochrome P450 (CYP) 3A4, which is inactive against SARS‐CoV‐2 Mpro and increases simnotrelvir plasma concentrations by inhibiting CYP3A4‐mediated metabolism of simnotrelvir [17, 18]. The phase I clinical trial of simnotrelvir/ritonavir showed that both single and multiple oral doses of simotevir/ritonavir are well tolerated [15]. The phase IB clinical trial further proved its good tolerance, and the recommended dose of simnotrelvir/ritonavir was determined [19]. A multicenter, randomized, double‐blind, placebo‐controlled phase II/III clinical trial (SIM0417‐301) of 1208 subjects with mild‐to‐moderate COVID‐19 [13, 14] showed that compared with placebo, simnotrelvir/ritonavir significantly shortened the symptom duration of mild‐to‐moderate COVID‐19 patients and rapidly and substantially reduced SARS‐CoV‐2 viral load, with a decrease in viral load of about 96%, a decrease in nucleic acid conversion time of about 2.2 days, and a decrease in the time from the first dose to complete elimination of symptoms of about 1.5 days after the 5‐day course of treatment.

At present, there are few clinical studies on simnotrelvir/ritonavir. In our study, we found that, among the 115 patients, most patients (67.8%) had different numbers of high‐risk factors, including older age, immunodeficiency, obesity, and heavy smoking. Besides, a number of patients (46.1%) had more than two high‐risk factors, and 13.9% of patients had not received coronavirus vaccines, which made patients more susceptible to coronavirus infection. The baseline CT values of both groups were high, 29.8 (24.9, 31.5) in the simnotrelvir/ritonavir group and 26 (20.9, 32.7) in the nirmatrelvir/ritonavir group, which were higher than those in previous studies. The reason may be that the main epidemic strain in the region during this study period was Omicron BA.2.76. Studies have shown that the BA.2.76 variant has enhanced transmission and reduced pathogenicity compared with previous strains. Changes in the N gene's translation initiation region could impair the translation efficiency of the N gene [20].

Moderate to severe COVID‐19 patients exhibited higher mortality rates compared to mild cases [21, 22], which may be attributed to the development of ARDS, followed by an inflammatory cascade triggered by activated immune cells, ultimately leading to multiple organ failure and death [23]. Previous studies have demonstrated reduced hospitalization rates and mortality in outpatients or hospitalized patients with mild‐to‐moderate COVID‐19 after the use of antivirals [11, 24]. However, a certain proportion of patients will still progress to severe COVID‐19, and there are insufficient treatments for patients with severe COVID‐19 [25]. Studies have shown that when the immune system overreacts, immunosuppressive therapy may benefit severe patients (e.g., IL‐6 receptor antagonists, JAK inhibitors, and steroid hormones) [26]. In this study, all patients with severe COVID‐19 received both immunotherapy and oral antiviral therapy. The mortality rate of severe patients was 21.4% in the simnotrelvir/ritonavir group and 20.0% in the nirmatrelvir/ritonavir group. Although the number of cases was very small and not statistically significant, we can expect that the combination of immunotherapy and antiviral drug therapy will help severe patients. In this study, we aimed to compare the effectiveness of simnotrelvir/ritonavir and nirmatrelvir/ritonavir in treating moderate to severe COVID‐19. Overall, no significant differences were observed in the composite disease progression rate, mortality rate, or the transition from moderate to severe COVID‐19 between the simnotrelvir/ritonavir and nirmatrelvir/ritonavir groups. The mean time for SARS‐CoV‐2‐negative conversion and hospitalization duration were similar in both groups. However, simnotrelvir/ritonavir demonstrated a significantly shorter clinical recovery time in moderate patients. Although a placebo control group was not included, from a clinical outcome perspective, both simnotrelvir/ritonavir and nirmatrelvir/ritonavir exhibited the ability to delay disease progression, shorten hospitalization duration, and SARS‐CoV‐2‐negative conversion time, thereby benefiting patients with moderate to severe COVID‐19.

The incidence of ADRs in the simnotrelvir/ritonavir group (8.6%) was found to be comparable to that in the nirmatrelvir/ritonavir group (10.5%), with no significant difference observed between the two groups (*p* = 0.728). Following symptomatic treatment, the adverse reactions showed improvement, and no severe adverse events necessitated discontinuation of the medication. These findings indicate that patients generally tolerate simnotrelvir/ritonavir.

This study has some limitations. This study showed that the time for hospitalization of patients with moderate COVID‐19 was significantly shorter in the simnotrelvir/ritonavir group than in the nirmatrelvir/ritonavir group. Based on the small cohort in this study, this result may have certain limitations, and a large‐sample study is urgently needed to further evaluate whether there is a significant difference in hospital stays between the two. Because of the short marketing time of simnotrelvir/ritonavir, and many considerations such as comorbidities and drug interactions when clinicians choose antiviral drugs, we could not further collect case data from a larger number of samples. This study is a retrospective, single‐center study, which has not been prospectively validated at this stage. In addition, considering that the study population was moderate to severe COVID‐19 patients, most clinicians would choose to use antiviral drugs, and it was difficult to collect the cohort without antiviral drugs in a single center, so we have not included data on patients without the use of antiviral drugs. The observed difference in the median days from symptom onset to medication initiation may represent a confounding factor, potentially influenced by variations in initial outpatient visits or the small sample size. In addition, there are many factors influencing the prognosis and outcome of COVID‐19, and the influence of confounding factors cannot be ruled out. A multicenter, large‐sample clinical study is needed to obtain more reliable study results subsequently. Future research directions: a prospective randomized controlled trial, increase the number of enrolled cases, actively carry out multicenter joint clinical studies, add a placebo‐controlled group based on the current research protocol, and further evaluate the effectiveness and safety of simnotrelvir/ritonavir in patients with moderate to severe COVID‐19.

This study compared the effectiveness and safety of simnotrelvir/ritonavir and nirmatrelvir/ritonavir in the treatment of moderate to severe COVID‐19. The findings suggest that both antivirals demonstrate comparable safety profiles, with nirmatrelvir/ritonavir showing slightly better outcomes in some measures. These results provide some insights into the relative performance of these treatments and inform clinical decision‐making.

## Author Contributions

**Xin Chen:** data curation, writing – original draft. **Xiao‐Qing Lin:** data curation, methodology, writing – original draft. **Fang Cheng:** data curation. **Shi‐Lin Zheng:** data curation. **Qiang Zhang:** data curation. **Te Wu:** data curation. **Xian‐Gao Jiang:** conceptualization, project administration, writing – review and editing. **Ji‐Chan Shi:** conceptualization, project administration, supervision, validation, writing – review and editing.

## Ethics Statement

This study was approved by the Ethics Committee of Wenzhou Central Hospital (L2023‐04‐079). All patients were informed and agreed to participate in this study.

## Conflicts of Interest

The authors declare no conflicts of interest.

## Supporting information

Supporting information.

## Data Availability

All raw data and code are available upon request.
