# Supplementary material for: Effectiveness and Safety of Simnotrelvir/Ritonavir and Nirmatrelvir/Ritonavir in the Treatment of Moderate to Severe COVID‐19
Source: Immun Inflamm Dis. 2025 Apr 14;13(4):e70174. doi: 10.1002/iid3.70174 (PMC11995419; doi:10.1002/iid3.70174)
Supplement: Supplementary file 1 — Supporting information. [file IID3-13-e70174-s001.docx]

**Supplementary Table** 1. **Tests of Normality**

|  | Kolmogorov-Smirnov^a^ | | | Shapiro-Wilk | | |
| --- | --- | --- | --- | --- | --- | --- |
|  | Statistic | df | Sig. | Statistic | df | Sig. |
| Age (years) | 0.142 | 115 | ＜0.001 | 0.924 | 115 | ＜0.001 |
| Time-1 (Days) | 0.160 | 115 | ＜0.001 | 0.909 | 115 | ＜0.001 |
| Time-2 (Days) | 0.278 | 115 | ＜0.001 | 0.697 | 115 | ＜0.001 |
| Symptom scores | 0.201 | 115 | ＜0.001 | 0.915 | 115 | ＜0.001 |
| CT value | 0.115 | 115 | ＜0.001 | 0.972 | 115 | 0.015 |
| ORF1-a/b gene | 0.103 | 115 | 0.004 | 0.977 | 115 | 0.046 |
| N gene | 0.107 | 115 | 0.003 | 0.973 | 115 | 0.020 |
| Time to sustained recovery(Days) | 0.211 | 115 | ＜0.001 | 0.744 | 115 | ＜0.001 |
| Time to negative conversion of antigen or nucleic acid(Days) | 0.280 | 115 | ＜0.001 | 0.661 | 115 | ＜0.001 |
| a. Lilliefors Significance Correction | | | | | | |

Note. Sample size > 50, referring to Kolmogorov-Smirnov, the results suggest that all P ＜ 0.05, and the variables do not conform to normal distribution.

**Supplementary Table2. Distribution of high-risk factors for COVID-19**

| Variables | Categories | Total(N=115) | | P | Moderate (N=91) | | P | Severe(N=24) | | p |
| --- | --- | --- | --- | --- | --- | --- | --- | --- | --- | --- |
|  |  | Simnotrelvir/  ritonavir  group(N=58) | Nirmatrelvir/  ritonavir  group(N=57) |  | Simnotrelvir/  ritonavir  group（N=44） | Nirmatrelvir/  ritonavir group（N=47） |  | Simnotrelvir/  ritonavir  group（N=14） | Nirmatrelvir/  ritonavir  group（N=10） |  |
|  |  | M(P25,P75),n(%) | |  | M(P25,P75),n(%) | |  | M(P25,P75),n(%) | |  |
| COVID-19 High-Risk Factors (Yes) |  |  |  |  |  |  |  |  |  |  |
|  | Cardiovascular Disease | 20(34.5) | 15(26.3) | 0.419 | 11（25.0） | 8（17.0） | 0.441 | 9(64.3) | 7(70.0) | 1.000 |
|  | Chronic Lung Disease | 3（5.2） | 0（0.0） | 0.243 | 0(100) | 0(100) | / | 3(21.4) | 0(0.0) | 0.239 |
|  | Diabetes | 11(19.0) | 6(10.5) | 0.294 | 5(11.4) | 4(8.5) | 0.734 | 6(42.9) | 2(20.0) | 0.388 |
|  | Chronic liver disease | 0(0.0) | 1(1.8) | 0.496 | 0(0.0) | 1(2.1) | 1.000 | 0(0) | 0(0) | / |
|  | Chronic kidney disease and patients on maintenance dialysis | 0(0.0) | 0(0.0) | / | 0(0.0) | 0(0.0) | / | 0(0) | 0(0) | / |
|  | Tumors | 17(29.3) | 2(3.5) | ＜0.001 | 12(27.3) | 2(4.3) | 0.003 | 5(35.7) | 0(0.0) | 0.053 |
|  | Immunodeficiency | 7（12.1） | 2（3.5） | 0.162 | 4(9.1) | 2(4.3) | 0.425 | 3(21.4) | 0(0.0) | 0.239 |
|  | Heavy smokers | 5（8.6） | 1(1.8) | 0.206 | 4(9.1) | 0(0.0) | 0.051 | 1(7.1) | 1(10.0) | 1.000 |
|  | BMI >30 | 3（5.2） | 2（3.5） | 1.000 | 2(4.5) | 1(2.1) | 0.608 | 1(7.1) | 1(10.0) | 1.000 |
|  | Age ≥65Y | 35(60.3) | 30(52.6) | 0.454 | 24(54.5) | 21(44.7) | 0.404 | 11(78.6) | 9(90.0) | 0.615 |
